# Influence of precipitation on psychiatric emergency care visits

**DOI:** 10.1192/j.eurpsy.2021.1874

**Published:** 2021-08-13

**Authors:** D. Hernández-Calle, M.F. Bravo-Ortiz

**Affiliations:** Psychiatry, Hospital Universitario La Paz, Madrid, Spain

**Keywords:** Emergency Care, Suicide

## Abstract

**Introduction:**

Psychiatric emergency care visits have been associated to several climate variables. However, the influence of precipitation has been understudied

**Objectives:**

To study the association between precipitation and number of emergency care psychiatric visits.

**Methods:**

Daily urgency visits were extracted from electronic medical records of Hospital Universitario La Paz from 1st January 2019 to 31th December 2019. Precipitation data (measured as accumulated litres per square meter) was obtained from a local climate station. Spearman correlation was estimated.

**Results:**

The Spearman correlation coefficient was -0.02(p = 0.80).

**Conclusions:**

Precipitation was not associated to number of emergency visits.

**Disclosure:**

No significant relationships.

